# Range expansion of muskox lungworms track rapid arctic warming: implications for geographic colonization under climate forcing

**DOI:** 10.1038/s41598-020-74358-5

**Published:** 2020-10-14

**Authors:** Pratap Kafle, Peter Peller, Alessandro Massolo, Eric Hoberg, Lisa-Marie Leclerc, Matilde Tomaselli, Susan Kutz

**Affiliations:** 1 Department of Ecosystem and Public Health, Faculty of Veterinary Medicine, University of Calgary, 3330 Hospital Drive NW, Calgary, AB T2N 4N1 Canada; 2 Spatial and Numeric Data Services, University of Calgary, 410 University Ct NW, Calgary, AB T2N 1N4 Canada; 3 Department of Biology, University of Pisa, Via Volta 6, 56126 Pisa, Italy; 4 Department of Biology and Museum of Southwestern Biology, University of New Mexico, Albuquerque, NM 87131 USA; 5 Department of Pathobiological Sciences, School Veterinary Medicine, University of Wisconsin, Madison, WI 53706 USA; 6 Department of Environment, Government of Nunavut, Kugluktuk, NU X0B 0E0 Canada; 7 Canadian High Arctic Research Station, Polar Knowledge Canada, 1 Uvajuq Road, Cambridge Bay, NU X0B 0C0 Canada; 8 Present Address: Western College of Veterinary Medicine, University of Saskatchewan, 52 Campus Dr., Saskatoon, SK S7N 5B4 Canada

**Keywords:** Climate-change ecology, Ecosystem ecology, Biogeography, Ecology, Ecology

## Abstract

Rapid climate warming in the Arctic results in multifaceted disruption of biodiversity, faunal structure, and ecosystem health. Hypotheses have linked range expansion and emergence of parasites and diseases to accelerating warming globally but empirical studies demonstrating causality are rare. Using historical data and recent surveys as baselines, we explored climatological drivers for Arctic warming as determinants of range expansion for two temperature-dependent lungworms, *Umingmakstrongylus pallikuukensis* and *Varestrongylus eleguneniensis*, of muskoxen (*Ovibos moschatus*) and caribou (*Rangifer tarandus*), in the Canadian Arctic Archipelago from 1980 through 2017. Our field data shows a substantial northward shift of the northern edge of the range for both parasites and increased abundance across the expanded ranges during the last decade. Mechanistic models parameterized with parasites’ thermal requirements demonstrated that geographical colonization tracked spatial expansion of permissive environments, with a temporal lag. Subtle differences in life histories, thermal requirements of closely related parasites, climate oscillations and shifting thermal balances across environments influence faunal assembly and biodiversity. Our findings support that persistence of host-parasite assemblages reflects capacities of parasites to utilize host and environmental resources in an ecological arena of fluctuating opportunity (alternating trends in exploration and exploitation) driving shifting boundaries for distribution across spatial and temporal scales.

## Introduction

The unprecedented rate of warming in the Arctic is having repercussions across terrestrial and marine systems, leading to changes in ecosystem structure and function^1,2^, including shifting diversity and dynamics of wildlife disease/pathogens^3,4^. Increased temperatures, resulting in the northward shift of isotherms, are altering the zones of climatic suitability, or permissive environments, for pathogens and parasites, and this is being increasingly linked to invasions, range shifts and disease outbreaks^5–9^. Empirical observations and mechanistic models from the Arctic were the first to establish an unequivocal and direct link between climate warming and changing host–pathogen dynamics for nematode parasites, resulting in alterations in pathogen ecology and distribution^7,8^.

In the Arctic, understanding the outcomes of climate-driven perturbations on host–pathogen interactions is essential to a synoptic view of a biosphere in accelerating transition. Pathogens are fundamental components of ecosystems and understanding host–pathogen dynamics is critical for conserving healthy wildlife populations which are the foundations for food security and well-being of northern communities and Indigenous peoples^10^. The Arctic offers a unique landscape for testing hypotheses and models on host-parasite dynamics because of its relative simplicity, minimal confounding variables, and rapid rate of change^11^. Studying responses of Arctic host-parasite systems to climate warming provides broader insights on how climate oscillations on evolutionary to ecological time scales have historically determined and continue to influence the dynamics, evolution, and biogeography of parasites and the biosphere^12,13^.

**Figure 1:**
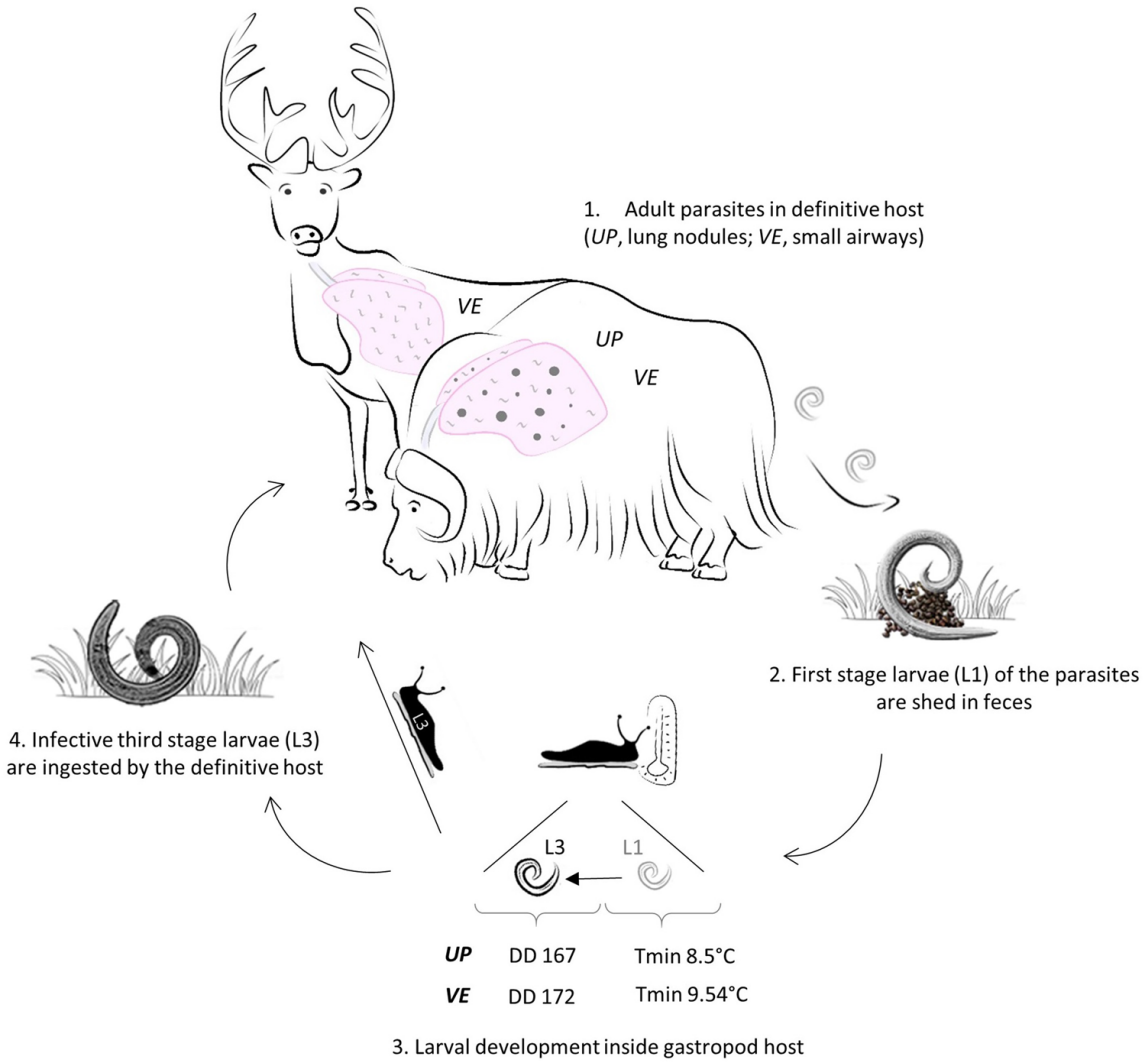
The lifecycle of the protostrongylid nematodes *Umingmakstrongylus pallikuukensis* and *Varestrongylus eleguneniensis*. The lifecycle is indirect, involving gastropods as intermediate hosts (IH) where first stage larvae (L1) develop into infective third stage larvae (L3), the rate of which is determined by gastropod temperature. The L3 are ingested by the definitive hosts either as larvae that have emerged from the IH into the environment, or while still inside the gastropods. *Umingmakstrongylus pallikuukensis *(*UP*) and *Varestrongylus eleguneniensis *(*VE*) follow this life cycle pattern but differ in key thermal parameters (lower developmental threshold—*Tmin*, development degree-days—*DD*) and other life history features (e.g., host specificity, fecundity, life span)^17–19^. *Umingmakstrongylus pallikuukensis* is specific to muskoxen, is long-lived, and highly fecund, whereas *VE* can infect both caribou and muskoxen, has a shorter lifespan and much lower fecundity. (Artwork: M. Tomaselli).

*Umingmakstrongylus pallikuukensis* (hereafter referred to as *UP*) and *Varestrongylus eleguneniensis* (hereafter referred to as *VE*) are protostrongylid nematodes of muskoxen (*Ovibos moschatus*) and caribou (*Rangifer tarandus*), two important species which have experienced recent local to global population declines^14,15^. Protostrongylid nematodes have indirect temperature-dependent lifecycles (Fig. 1): larval development, and thus geographic ranges of this group of parasites, are limited by species specific climatic envelopes and availability of suitable thermal habitats and intermediate hosts that facilitate or limit the persistence of host-parasite assemblages. As model systems, these nematodes provide a direct pathway to understanding the impacts of climate change on host-parasite systems^16^.

The muskox lungworm, *UP,* has served as the ‘poster child’ for the effect of climate warming on parasites in the Arctic, and was the first helminth parasite globally in which changes in development and geographic distribution were linked to patterns of accelerating warming^7,8^. High temperature anomalies in the late 1980s, followed by sustained above-average temperatures, resulted in a tipping point for this parasite leading to a shift from a multi-year to a single year lifecycle in the core of its range on the central Arctic mainland^7^. More recently, continued and accelerating warming in Arctic environments has been associated with northward geographic expansion with invasion and establishment of *UP* and of *VE,* a related lungworm that infects both muskoxen and caribou*,* onto Victoria Island, in the Canadian Arctic Archipelago^8^. The recent invasion of these two parasites, which have similar life histories but different ecological characteristics and lifecycle dynamics (Fig. 1), provides a unique opportunity to assess, in real time, the effects of incremental climate warming, short-term thermal oscillations and the implications for tipping points^7^, and thresholds on parasite invasion (e.g.,^9,13^ ). In this study, we describe spatial and temporal patterns of abundance of *UP* and *VE* in the Arctic from 1980 to 2017, including historical distribution and northward spread. We then compare our observational data to the predicted thermal niches of *UP* and *VE* for 1980–2017, determined using a mechanistic model based on development degree-days inside the gastropod intermediate host. We contribute to broader insights about climate warming and oscillation across temporal and spatial scales and processes in the biosphere that determine the distribution of pathogens and disease.

## Materials and methods

### Study area

**Figure 2:**
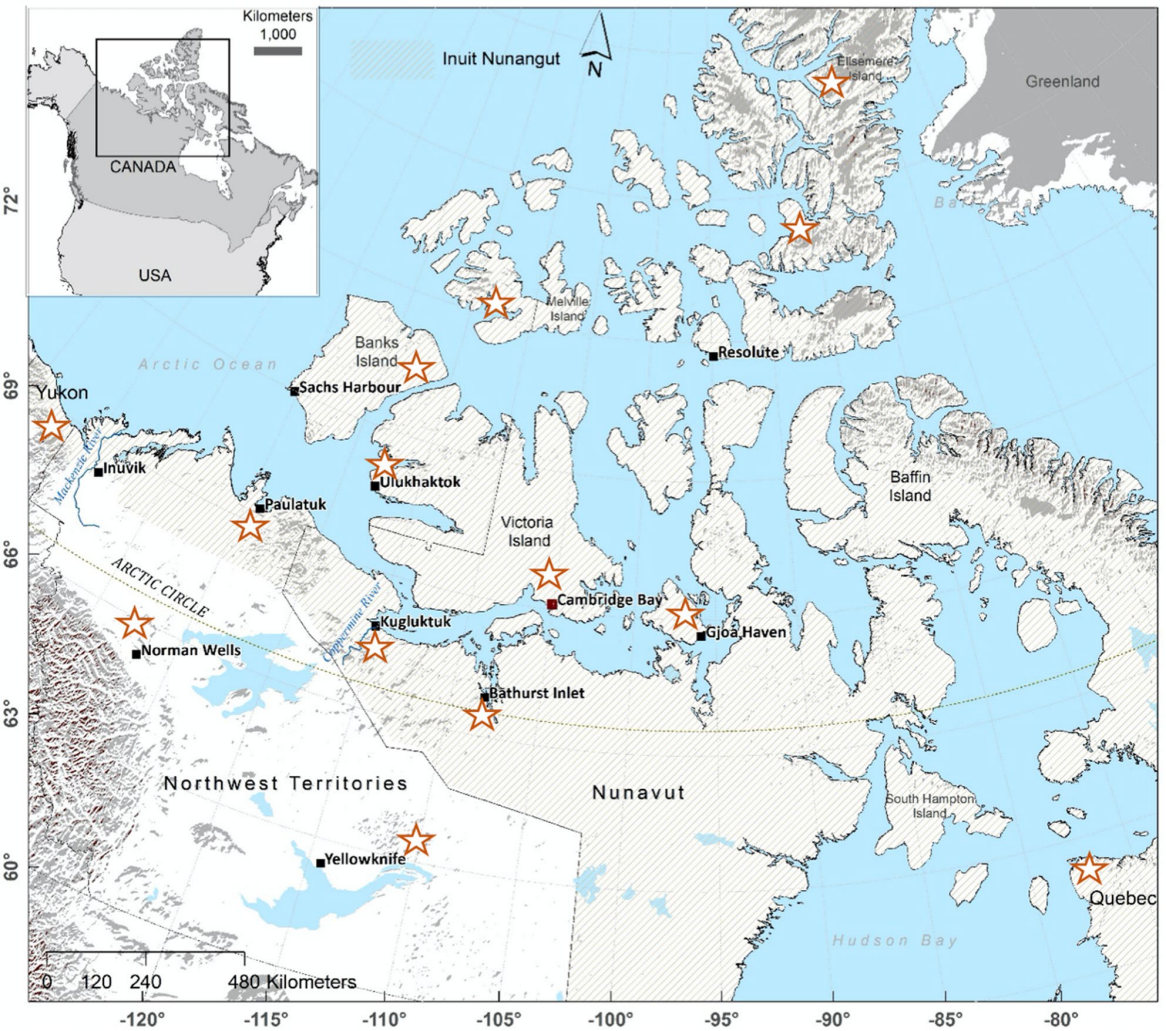
Map showing the study area and general localities (**☆**) where the muskox fecal samples were collected between 2013 and 2017. Fecal surveys were carried out mainly within Inuit Nunagut region, as well as in the Sahtu (Norman Wells) and North Slave (east of Yellowknife) areas of Northwest Territories, and northern Yukon (west of Inuvik). The map was generated in ArcGIS software version 10.6 (ESRI 2011, ArcGIS Desktop: Release 10. Redlands, CA, Environmental Systems Research Institute, https://www.esri.com/en-us/home).

The study area mainly covers the Arctic and Subarctic regions of the Inuit Nunangat in Canada, with the Inuit communities of Ulukhaktok, Northwest Territories, and Kugluktuk and Cambridge Bay, Nunavut in the core study area (Fig. 2). This region has experienced rapid environmental changes in the last few decades: e.g., the annual mean temperature north of 60º north latitude increased by 2.3 °C (likely range 1.7–3.0 °C) from 1948 to 2016. This is roughly three times the global mean warming rate of 0.8 °C^20,21^. Climate models predict an increasing rate of future Arctic warming, with a projected rise in annual temperature by the end of century (2081–2100 avg) between 2.1 (RCP2.6) and 7.8 °C (RCP8.5)^21^.

Muskoxen and caribou are distributed throughout much of northern Canada, although their abundance varies^14,15^. Barren-ground caribou herds occur on the mainland, and the Dolphin and Union caribou herd, a unique ecotype, winters on the mainland and seasonally migrates to calve and summer on Victoria Island^14^. The Committee on the Status of Endangered Wildlife in Canada has recommended the Dolphin and Union caribou as Endangered^14^. The muskox populations of Victoria and Banks Islands have declined by greater than 50–70% since the early 2000s while the populations on the adjacent mainland have remained relatively stable^15^.

### Field and laboratory methods

We drew on the literature, archival data (since the 1980s), along with field-survey and monitoring based on new collections and fecal sampling to determine the historical and recent distribution of *UP* and *VE*. Survey data for 1988–2012 (historical data) were derived from previously published studies^8,22,23^ and data for the recent period, 2013–2017, originated from new sampling and collections following standard protocols.

Between April 2013 and December 2017, 1380 muskox fecal samples were collected from various regions of the Canadian Arctic and Subarctic. Samples were collected opportunistically by regional biologists and field scientists during fieldwork, and by hunters, guides and outfitters during sport, commercial and subsistence hunts. Samples were also collected strategically at targeted sites, with an attempt to detect the northern and eastern edges of the parasites’ ranges. Samples were frozen upon collection and sent to the University of Calgary for further analyses. All the samples were stored at -20 °C and analyzed within 1 to 6 months of collection.

Fecal samples were analyzed using the modified beaker Baermann technique^24^. Briefly, 5 g (on average) of fecal pellets were placed in between the two layers of mesh and a cheesecloth and submerged in the beaker full of tap water for 24 h under light. First stage larvae (L1) were extracted and counted using total and aliquot counts (in heavy infections). Samples were run in pairs in areas of low larval abundance (northern and eastern edges of ranges) and the larval counts were averaged. Extracted L1 were identified to species using morphological keys^25–27^, counted, and quantified on a per gram basis (LPG). Voucher specimens of larvae determined as *UP* and *VE* have been held in ethanol and cryo-archived in museum collections of the Museum of Southwestern Biology, University of New Mexico, and databased in the Arctos platform (https://arctos.database.museum) [MSB:Para:31452 to MSB:Para:31458].

### Modelling

**Figure 3:**
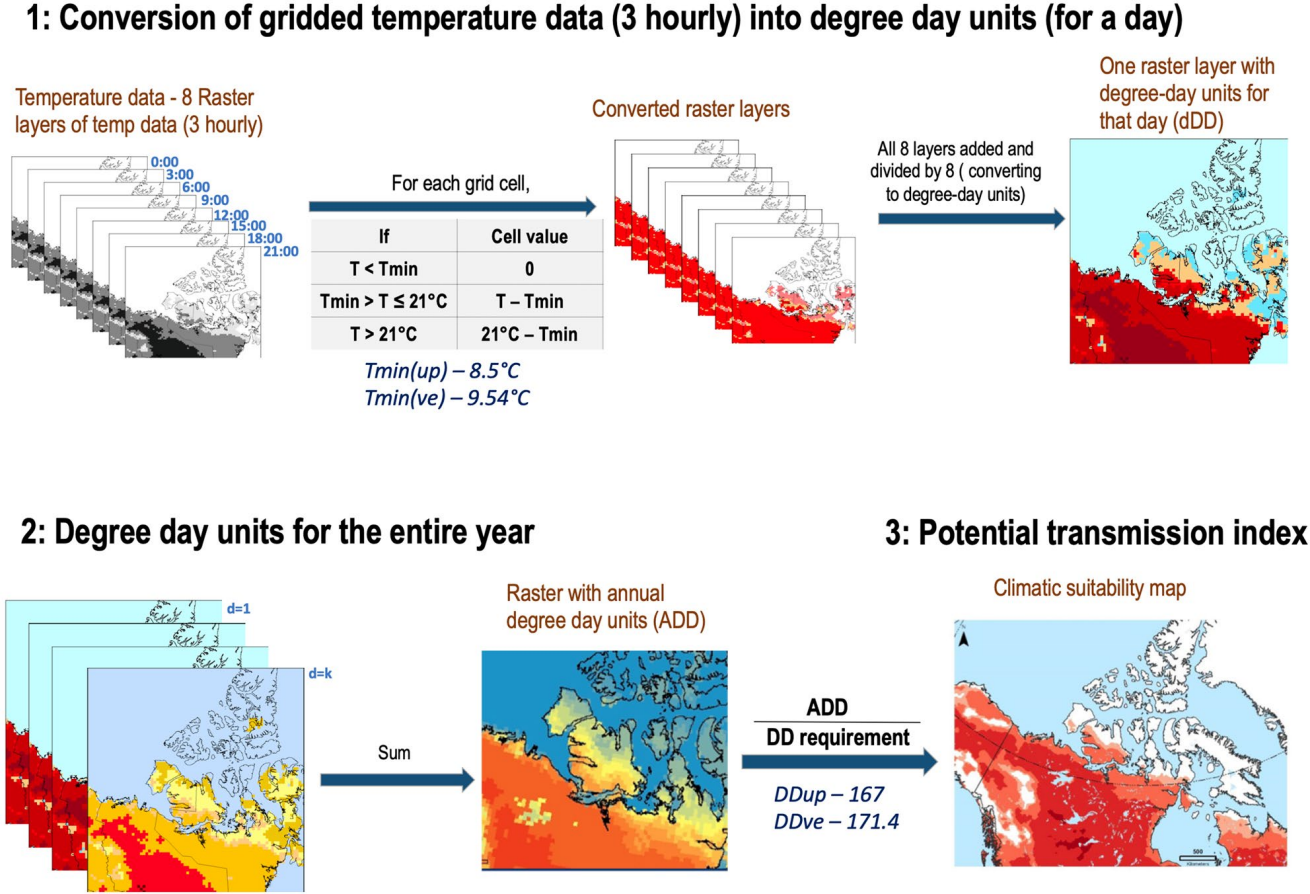
Modelling framework to generate the maps showing permissive thermal conditions for development. First, for each grid cells, degree-day (*DD*) units accumulated in a day (*dDD*) was determined by calculating the degree-units (*D*) for each time slice of the day (8 time slices per day). The value *D* for each time slice is calculated as the difference between the temperature at that time, or the upper temperature cut-off (21 °C), whichever is smaller, and the lower development threshold (*Tmin*). To calculate *dDD*, total *D* units in a day are summed and divided by 8 (see^31,32^). Finally, annual cumulative degree-days (*ADD*) for each year was derived by summing the *dDD* values for the entire year. The final step involved the conversion of the *ADD* units into potential transmission index (*PTI*) units by dividing the *ADD* units by the threshold *DD* units for each species. The maps were generated in ArcGIS software version 10.6 (ESRI 2011, ArcGIS Desktop: Release 10. Redlands, CA, Environmental Systems Research Institute, https://www.esri.com/en-us/home).

We parameterized a spatially explicit process-based mechanistic model (Degree-day model^28^) to determine annual accumulations of development degree-days (*ADD*) from 1980 through to 2017 and mapped the geographical extent of the thermal niches in which *UP* and *VE* could develop from an L1 to an infective L3 within a single summer (Fig. 3). For each cell in the gridded map, the temperature values were converted to *ADD* units following Eq. (1):

1 $$ADD=\frac{1}{8}{\sum }_{d=1}^{k}{\sum }_{h=i}^{8}\mathrm{max}(0, Th\left(d\right)-Tmin)$$

where *k* is the upper summation limit for the year. *T*_*h*_ is the temperature of the h-th hour of the day (we used 3 hourly temperature readings—0:00, 3:00, 6:00, …, 21:00, so 8 time slices/day) and *Tmin* is the minimum threshold temperature for development (8.5 °C for *UP*^29^ and 9.54 °C for *VE*^18^). *Th* > 21 °C were defaulted to 21 °C to account for behavioral thermoregulation by the slug intermediate host; slugs tend to seek microclimates at 21 °C when the outside temperature exceeds 21 °C^30^ and this cut-off value was previously validated in experimental field studies by Kutz et al^7^.

The potential thermal niches were delimited by the geographic areas where the minimum thermal conditions for complete development of L1 to infective L3 could be obtained during a single summer: 167 degree-days (*DD*) above the developmental threshold (*Tmin*) of 8.5 °C for *UP*^29^ and 171.4 *DD* above the threshold of 9.54 °C for *VE*^18^ (Fig. 3).

The temperature data were derived from the North American Regional Reanalysis (NARR)^33^. The NARR is a state-of-the-art climate model reanalysis that provides fine spatial (grid resolution of approximately 0.3-degree (32 km) at the lowest latitude) and temporal (3 hourly) resolution across North America. Thermal parameters; lower development threshold (*Tmin*) and development degree-days (*DD*) for the two species were derived from our earlier work^18,29^. Calculation of degree-day units was based on horizontal cut-off method^34^ which assumes that development continues at a constant rate at temperatures above the upper threshold. The upper-temperature limit of 21 °C was used for both species, which is based on the behavioural thermoregulation by the main gastropod intermediate host, *Deroceras laeve*.

We used a Parasite Transmission Index (*PTI*), derived by dividing the *DD* values by the threshold number of *DD* required by the parasites for complete development into infective stage larvae to standardize comparisons between the two species, which have different heating requirements. A *PTI* below 1 indicates insufficient summer heating or *DD* for development from L1 to L3 in gastropod intermediate hosts, a *PTI* of 1 indicates enough *DD* for development and a PTI of 2 means double the number of required degree days have been accumulated. The total area of suitable thermal conditions for each year was estimated by calculating the entire area of the grid cells where *PTI* ≥ 1. To determine the trend in *ADD* accumulation around Cambridge Bay (Victoria Island), the mean of *ADD* estimates for the grid cells that lie within the 50 km radius of Cambridge Bay location (69 ${}^{\circ }$ 06′29" N, 105 ${}^{\circ }$ 08′18" W) (land only) were extracted from 1980 through to 2017. The analysis was performed in ArcGIS software version 10.6 (ESRI 2011, ArcGIS Desktop: Release 10. Redlands, CA, Environmental Systems Research Institute), using the *Spatial analyst* toolbox.

We acknowledge that *UP* can overwinter in gastropod hosts and resume development the following year^35^. It is unknown if *VE* has a similar life history. For simplicity, we modelled for single year development but recognized that for *UP* at least, and probably *VE*, this is a conservative estimate of the thermal niche.

### Statistical analyses

Statistical analyses were performed using R statistical software, version 3.4.2^36^. Prevalence for each parasite species was quantified as the percent of fecal samples with larvae of the specific parasite, the intensity of infection as the median of the larval counts (LPG) of infected hosts only, and abundance as the mean larval counts from all individuals sampled^37^.

A generalized linear model (negative binomial model, log link) was fitted to determine if the intensity of infection for both parasite species in muskoxen around Cambridge Bay changed between 2009 and 2017. The response variable was the number of larvae counted in 5 gm of feces, and the explanatory variables were year and species. The nonparametric Mann–Kendall (*MK*) trend test (significance level, 5%) was applied to identify the trends in *ADD* accumulated around Cambridge Bay from 1980 through to 2017.

## Results

### Range expansion and changes in abundance

**Figure 4:**
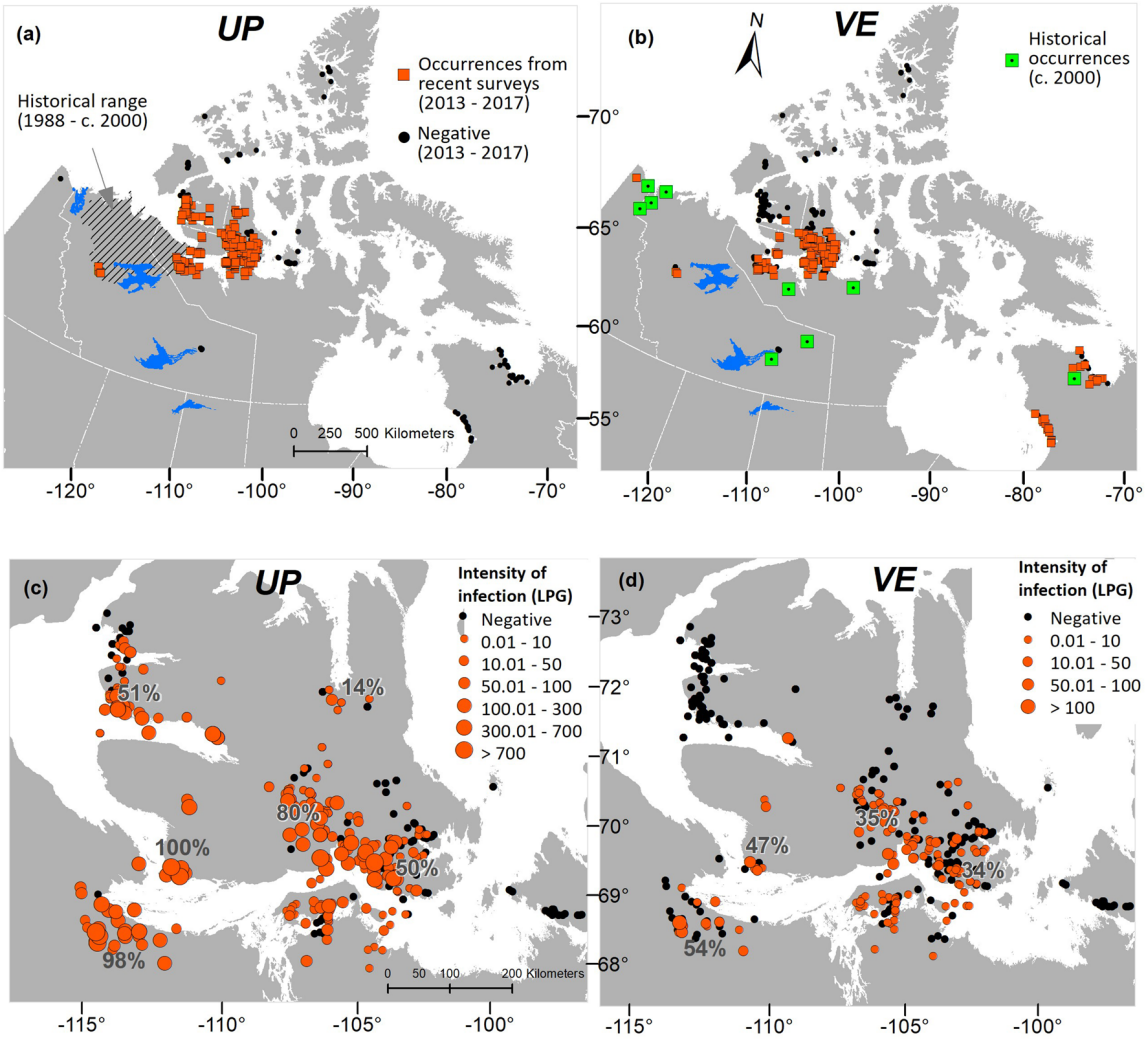
Northward expansion of the geographic ranges of *Umingmakstrongylus pallikuukensis* (UP) and *Varestrongylus eleguneniensis* (VE). (**a,b**) Maps show the historical and current ranges of *UP* and *VE* in muskoxen. The historical range for *UP* was restricted to the mainland and bounded by the Mackenzie River in the west and the Coppermine River in the east (see methods, Fig. 2), prevalence in muskoxen approached 100% in this area^16,23^. The historical occurrences for *VE* were derived from Kutz, et al*.*^22^ and Verocai et al*.*^38^. Point occurrences are provided for *VE* as there were fewer surveys, however, the parasites were likely distributed continuously across the mainland in their migratory caribou hosts^38^. (**c**,**d**) The 2013–2017 range and intensity of infection (LPG, larval counts per gram of feces) for *UP* and *VE* on Victoria Island and the adjacent mainland. The text overlaid on the maps represent the prevalence of the parasites in muskoxen during this time. These figures show a clear latitudinal gradient in prevalence and infection intensities, indicating the northern invasion trajectory, as well as species specific differences in those parameters. The maps were generated in ArcGIS software version 10.6 (ESRI 2011, ArcGIS Desktop: Release 10. Redlands, CA, Environmental Systems Research Institute, https://www.esri.com/en-us/home).

Populations of both *UP* and *VE* have undergone substantial geographic expansion, especially in the last decade (2009–2017) (Fig. 4a,b; Supplementary table 1) based on geographically extensive and site intensive surveys of muskoxen (larvae in fecal samples) across the Canadian Arctic, with a main focus on Victoria Island and the adjacent mainland (Fig. 2). Geographic colonization is most notable onto and across Victoria Island, (Northwest Territories and Nunavut), where *UP* is now found at least 400 km and *VE* at least 200 km north of the ranges previously reported by Kutz et al*.*^8^. Additionally, *UP* has expanded the eastern limit of its mainland range by approximately 500 km (Fig. 4). In contrast, *VE* was already broadly distributed across the temperate, subarctic and arctic mainland of North America^22,25,38,39^. Based on surveys and monitoring during the past decade, we did not detect either parasite on any islands of the Arctic Archipelago other than Victoria Island; *UP* was also absent from Quebec and the eastern Nunavut mainland (Fig. 4).

**Figure 5:**
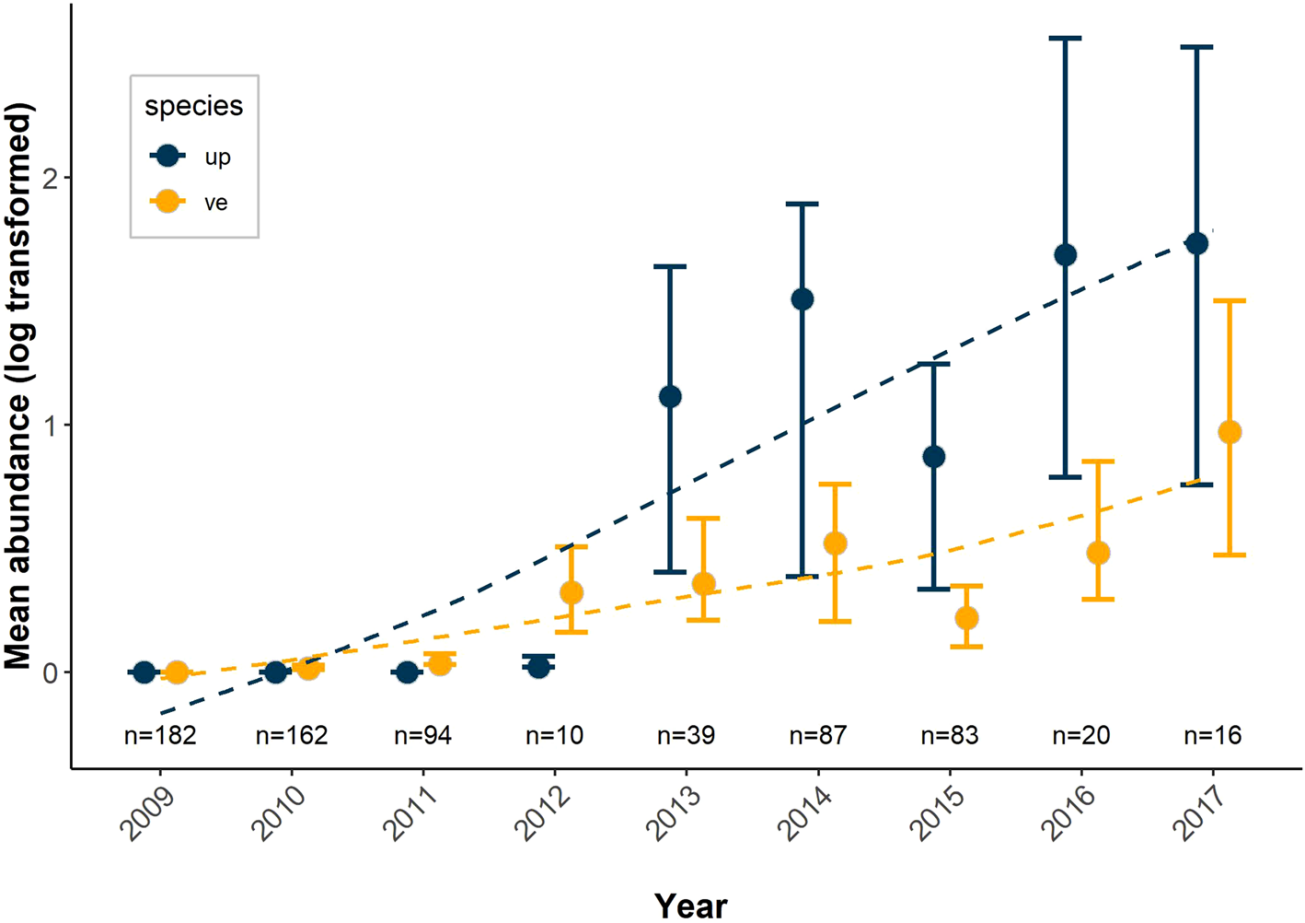
The increase in the larval abundance for *UP* and *VE* where muskoxen were continually sampled near Cambridge Bay from 2009 to 2017. The error bars represent the 95% confidence interval around the mean larvae per gram count (log transformed) of all sampled muskoxen. The lines represent the fit of *loess* regression. Abundance data from 2009 to 2012 were derived from Kutz et al.^8^.

Near Cambridge Bay, Victoria Island, where muskoxen were monitored annually, we observed a rapid increase in abundance of both parasites following the initial detection of *VE* in 2010 and *UP* in 2012. Although *UP* was detected in the region later, the rate of increase in abundance of this parasite was significantly higher than that of *VE*. (Fig. 5, Supplementary Tables 2, 3).

### Northward expansion of the parasites’ thermally suitable areas.

**Figure 6:**
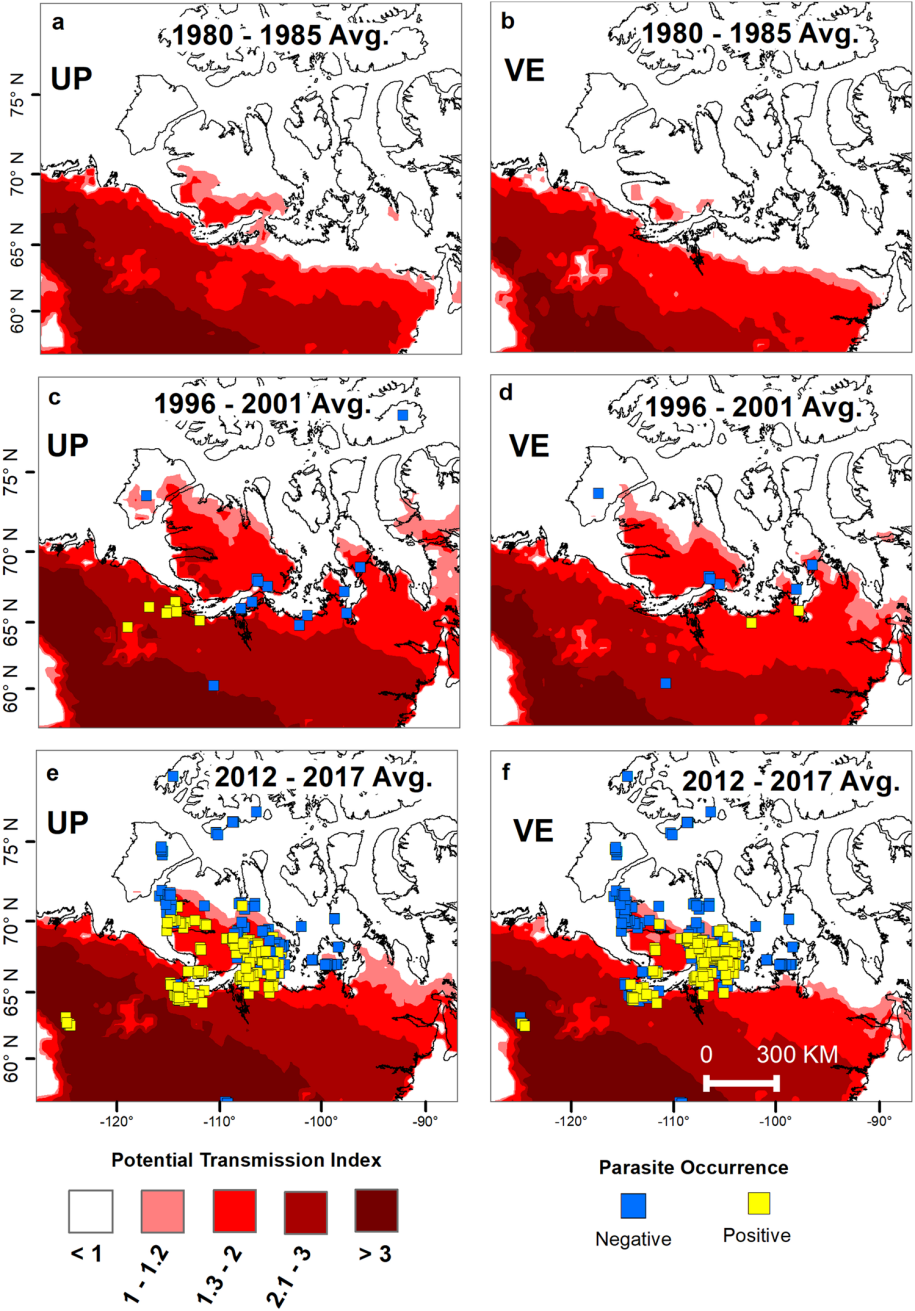
Northward expansion of the zones of suitable climate, based on parasite species specific accumulated degree days, from 1980 through to 2017. (**a–f**) Maps are showing the geographic extent of the thermal niches (represented by red shaded areas, from above 1 to darker red for values above 3 units of PTI) of the northern range of *UP* and *VE* during three six-year periods 1980–1985, 1996–2001, and 2012–2017. Darker shades of red indicate a greater number of accumulated degree days. The maps are produced by modelling degree-day accumulation for the two parasites using the 2 m air temperature (3 hourly) North American Regional Reanalysis (NARR) dataset by the National Centers for Environmental Prediction (NCEP)^33^. The average DD accumulated in six-year intervals, ten years apart, are displayed. Squares represent the known presence or absence of each parasite during the respective time frames. In maps **a** and **b**, no occurrence points are available as no surveys were done during that period. However, neither *UP* nor *VE* were detected on Victoria Island until 2008 and 2010, respectively, and neither have been found on any other arctic island despite targeted sampling since the late 1990s, thus they are both presumed to have been absent from the arctic archipelago in the 1980s. Occurrence points on maps **c** and **d** were obtained from published literature and our data. The maps were generated in ArcGIS software version 10.6 (ESRI 2011, ArcGIS Desktop: Release 10. Redlands, CA, Environmental Systems Research Institute, https://www.esri.com/en-us/home).

We found that the geographic area thermally suitable for the development and persistence of *UP* and *VE*, from landscape to regional scales, has expanded substantially on northward and eastward trajectories since the 1980s. This is represented in geographical space by the areas with the potential transmission index (*PTI*) values ≥ 1(Fig. 6). The 2012–2017 isolines of threshold *PTI* were considerably further north and east for both parasites compared to early 1980s (Fig. 6). The current northern limit of the observed distribution of *UP* based on field sampling is largely concordant with that of the northern extent of the modelled thermal niche for the same period. In contrast, the northern range limit of *VE* lags behind the northwestern extent of its thermal niche (Fig. 6f). The eastern range of *UP* on the mainland has also expanded consistent with the expansion of the thermally suitable areas; however, the extent of this expansion could not be determined because of insufficient sampling at the potential eastern range limits. *VE* is presumed to have been present historically in all mainland barren-ground caribou herds^22,38^, but sampling of muskoxen on the mainland east of the core study area was not adequate to determine distribution in this host.

Compared to the early 1980s, during the period 2012–2017, approximately 310,000 sq. km more of the Canadian Arctic became thermally suitable for development of *UP* and 420,000 sq. km for development of *VE* (Fig. 6)*.* However, there was considerable inter-annual variability in suitability. For example, in 1996–2001, the modelled thermally suitable range for both parasites was greater than that in 2012–2017. The summers of 1996, 1998, and 2000 were the warmest summers during our study period, and the high temperatures in those years are reflected in thermal suitability across a much greater geographic area during the period 1996–2001, including most of Victoria Island, the nearby islands and much of the eastern Canadian Arctic mainland (Fig. 6c, d). The apparent discrepancy between our results—greater predicted geographic range in the late 1990s compared to recent years—and the annual trends for increasing Arctic temperatures since the 1990s, are due to different warming patterns over time: while high temperatures in the late 1990s were driven by warmer summers, in the last decade they have been primarily driven by warmer winters^40,41^ (Supplementary Figs. 4, 5). This highlights the importance of seasonal and spatial patterns of warming and cooling on relatively short time scales of years to decades. The dynamics of ocean–atmosphere regimes, shifting between warm and cold cycles, such as El Niño Southern Oscillation (ENSO) or the Pacific Decadal Oscillation (PDO), may have pervasive effects in terrestrial systems and in predicting the outcomes of climate-drivers on the dynamics of host-parasite systems and the abundance, persistence and distribution of pathogens and disease (e.g.,^9,13^).

### Annual variation in thermal suitability for the two parasites around Cambridge Bay, NU

**Figure 7:**
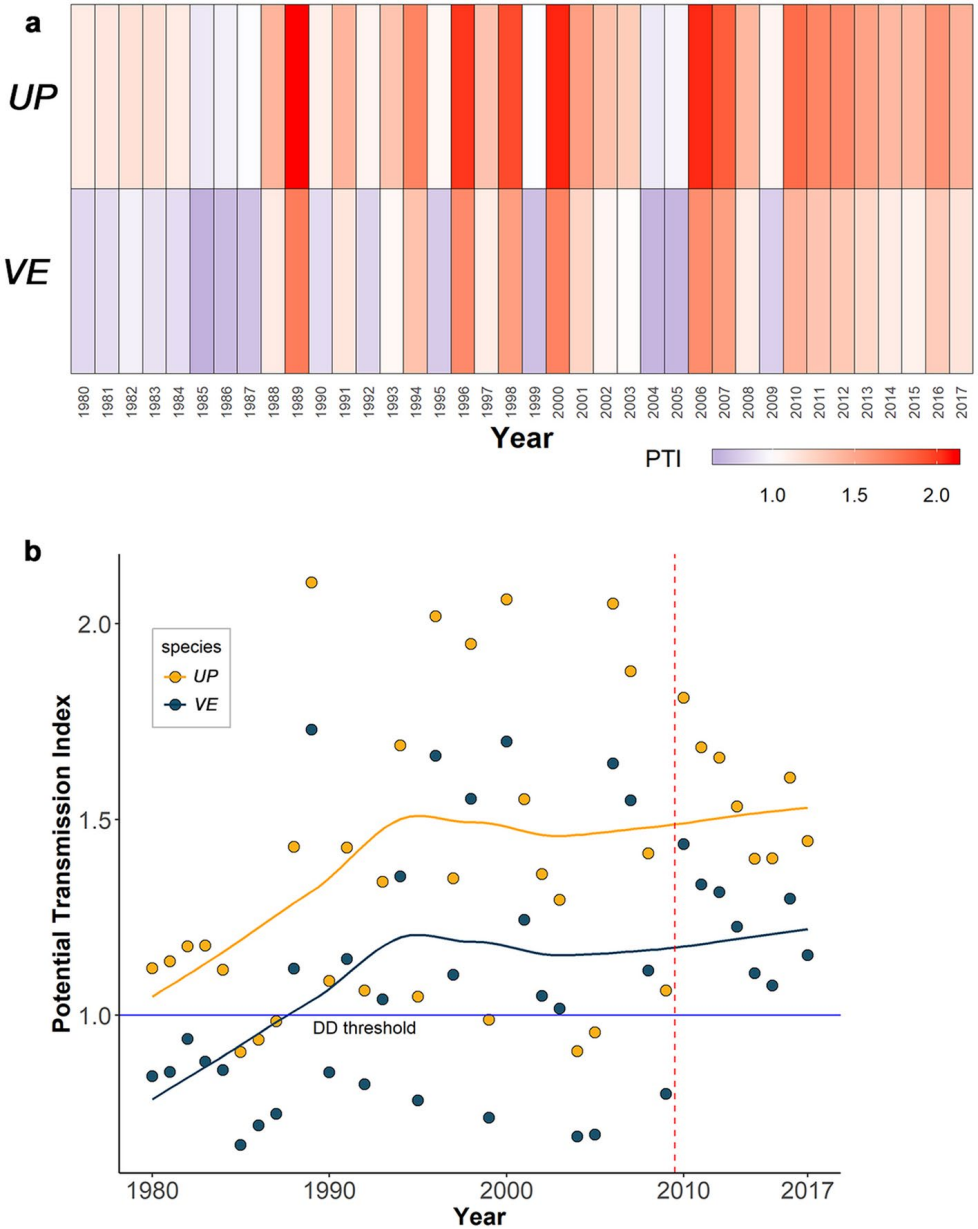
Annual variation in DD accumulation around Cambridge Bay from 1980 to 2017. (**a**) Heatmap and (**b**) graph showing the trend and variability in the potential transmission index for *UP* and *VE* within 50 km of Cambridge Bay from 1980 through to 2017. The PTI < 1 (blue colour in the heatmap) indicates unsuitable thermal conditions for L1–L3 development in a single summer. In the graph, the lines represent the fit of *loess* regression (span 0.4). The vertical dotted red line represents the year 2009 after which the PTI for both parasites was consistently > 1.

For both parasites, significant positive trends in the annual *PTI* around Cambridge Bay (*UP*: Kendall’s Tau (*τ*) = 0.22, *p* = 0.04, Mann–Kendall’s statistic (*S*) = 157; *VE*: *τ* = 0.21, *p* = 0.05, S = 152) with high interannual variability (Coefficient of variation (*CV*) for *UP*: 25.7%, CV for *VE*: 28.4%) were observed from 1980 to 2017 (Fig. 7). Previous research by Kutz, et al.^7^ described 1988–89 as a temperature and developmental tipping point in the core of the *UP* range; also coinciding with a recognized major tipping point in the acceleration of warming at high latitudes^42^. This is also reflected in our data for Cambridge Bay where, prior to 1988, development of *UP* within a single year was unlikely, but most years following 1988 have been suitable. Near Cambridge Bay from 2000 to present, except for 2004 and 2005, all years have been suitable for the development of *UP* within a single year. The annual thermal pattern is similar for *VE*, however, because of the higher threshold for development and higher heating requirements of this parasite, there are fewer suitable years, and those have a lower PTI compared to *UP*. Both parasites consistently had a *PTI* > 1 from 2010–2017 (Fig. 7).

## Discussion

It is well accepted that climate warming can result in distributional shifts of species, including pathogens, in space and through time, directly by dissolving the climatic barriers, indirectly by impacting other related biological systems (e.g., vector dynamics, host movement), or by influencing both processes concurrently^9,43–46^. In polar areas, where historically a cold and unfavourable climate has arguably been the major limiting factor for most species occurrences, high rates of climate warming in the last few decades have resulted in faster and more widespread poleward movement of species and restriction of refugial zones^43,45,47–49^. Despite the rapid warming and predictions of range expansion and pathogen emergence, there are few empirical examples globally that unequivocally demonstrate temperature as a driver^3,7,50^.

Kovats et al.^51^ recommended certain minimum requirements be met before linking pathogen emergence to climate change. These included (i) the monitoring of the pathogen should be done with adequate spatial and temporal coverage, (ii) controlled lab and field-based studies should investigate the pathogen’s physiological and epidemiological response (including the direction of response) to the environmental variable, and (iii) a long-term assessment of climatic conditions (including variability) in the region of interest and its correlation with changing patterns of distribution and abundance are necessary to conclude that the climate change is driving the emergence of the pathogen. Our work meets these requirements and demonstrates a direct association between parasite range expansion in the Arctic and climate change, coherent with expectations from empirical data from the lab^18,29^, monitoring data from the field, and degree-day modelling. Hence, this work provides strong empirical evidence of climate mediated changes in host parasite dynamics and distribution in the Arctic.

*UP* and *VE* were detected on Victoria Island for the first time in 2008 (southwest corner) and 2010 (Cambridge Bay), respectively. Based on the results of fecal examinations and characteristics of muskox lung nodules (i.e., small dimension and absence of calcification), it was concluded these were recent invasion and establishment events^8^. Our data demonstrate that the prevalence and intensity of infection of both parasites on Victoria Island follow a latitudinal gradient, with both being more abundant at southern latitudes on the island compared to the northern edge of their ranges (Fig. 4a,d, Supplementary Table 1), establishing clear evidence of a trajectory of range expansion. Our data showing increasing trends in abundance of the two lungworms near Cambridge Bay on southeast Victoria Island, (Fig. 5, Supplementary Tables 2, 3), further support interpretations about recent geographic colonization and continuing range expansion of these parasites on the island. Although logistical constraints prevented ongoing systematic sampling across the area of expansion, the data available also suggest an increase in abundance over time across the new range. Both patterns, movement on a northward trajectory and increasing abundance of parasites, are consistent with a dynamic and changing thermal environment emergent from a regional tipping point documented in 1970s^42^.

Despite periodic suitable thermal conditions for establishment, neither parasite was detected on Victoria Island until the 2000s^8^. For *UP*, which has only muskoxen as a definitive host, establishment on the island may have been prevented by the limited frequency of movement of muskoxen between the mainland and Victoria Island. This parasite is long lived in muskoxen and has high fecundity, with the average infected muskox shedding literally millions of freeze and desiccation tolerant larvae/day^19^. Consequently, once established, it is likely to persist in a region for several years during periodic episodes of unsuitable thermal conditions^19,52^. It is possible that the warmer summers of the late 1990s would have supported establishment if the parasite had been moved to Victoria Island by muskoxen, or less likely, anthropogenically as fomites on contaminated equipment (skidoos, sleds, etc.) when people travel between the mainland and island^8^, or through predators (passed through gastrointestinal tract)^53^. While there is recent genetic evidence and local knowledge of muskox movement between Victoria Island and mainland^54^ this appears to have been relatively rare historically^55^.

In contrast, the establishment of *VE* on the island may have been more constrained by temperature and other life history characteristics of the parasite. Introduction of the parasite to the island could have occurred through the annual migration of the Dolphin and Union caribou herd, or by infrequent movement of mainland muskoxen, or barren-ground caribou, to the island. Presuming that Dolphin and Union caribou could be infected on the mainland during winter through third stage larvae on the vegetation^25,56^, *VE* may have been transported to Victoria Island regularly with the annual seasonal migration of these caribou between the island and the mainland^8,9,14^. Our models suggest that thermal conditions on the island were periodically suitable for parasite establishment over the last 37 years, and increasingly so since the late 1990s. Nevertheless, the higher thermal requirements for the development, the substantially shorter lifespan in caribou, and the low fecundity of *VE*^17^, may have prevented its establishment on the island until there were multiple consecutive years with suitable conditions to facilitate establishment and persistence (i.e., 2010–2017). Other mechanisms for *VE* to be introduced to the island exist. Traditional knowledge indicates that some Dolphin and Union caribou periodically over-summer on the mainland (A. Hanke, pers. comm) so summer infection with subsequent migration to the island the following year may be a source of introduction. Conversely, introduction to Victoria Island may have been through muskox movement, similar to that for *UP,* with subsequent spill-over to Dolphin and Union caribou on their summer range on the island^8^. Finally, sporadic movement onto the island of barren-ground caribou (as reported through traditional knowledge [Hanke, pers. Comm]), in which *VE* is endemic^38^, could also have introduced the parasite to the island. Establishing the introduction route for *VE* to the island and determining whether maintenance on the island is dependent on caribou or muskoxen alone, or both species together, will require elucidation through parasite genetic analyses and more complex transmission dynamics models.

Unique to our analyses, we were able to simultaneously study two phylogenetically related parasites with similar life histories but different thermal requirements. Initially, given that *VE* has two definitive hosts, one of which is a migratory caribou, we expected that this parasite would have expanded its range more quickly than *UP,* both because it had a higher potential host density (both muskoxen and caribou) and it could be moved more quickly across the landscape within its migratory caribou host*.* Our data showed the contrary, with *UP* leading the expansion front on Victoria Island. This is further supported by our degree-day models which showed that *PTI* of *VE* is consistently lower than that of *UP* for any given time and location, and the empirical data demonstrated that the observed distribution limit of *VE* lagged behind the spatial limit of its potential thermal niche. Whereas the higher thermal requirements of *VE* explain the lower *PTI*, the apparent lag of *VE* to fill its potential thermal niche is more likely related to its lower fecundity and a shorter lifespan.

These findings suggest that, at least in this system, the life history, as well as the physiological and thermal requirements of the parasites, may be stronger determinants of distribution and range expansion than host factors such as density and migration behaviour. A key component of the life cycle of these parasites that was not investigated was the ecology and distribution of the gastropod intermediate hosts. *Deroceras laeve,* the meadow slug, is considered the primary intermediate host species for both parasites^23,29^. This slug is present on the island at low densities^57^. Features of parasite species-specific development, survival, and emergence rates within *D. laeve*, as well as gastropod diversity, density and distribution relative to muskox and caribou habitat use, likely play a role in differential rates and patterns of range expansion. It is the thermal arena, however, that serves to determine parasite development and persistence in the environment and ultimately is the limiting factor that controls distribution.

We used a process-based mechanistic model using a thermal constant required for the development of the larval stages of the parasites (development degree-days: *DD*) to reveal the change in the geographical extents of the thermal niche for two invasive parasites over space and time, and to determine if the observed parasite range expansion could be explained by the ongoing Arctic warming. These models quickly demonstrated spatial and temporal differences in the parasites’ thermal niches, and different expansion rates between two parasite species that have generated new hypotheses. The results supported a tipping point in the late 1980s as proposed by Kutz et al.^7^, and identified a possible second ‘tipping point’ where intermittent suitability in annual temperatures have flipped to a phase of continuous suitability. This observation is consistent with the proposal for a *shifting thermal balance* driven by climatological conditions that serve to either limit or facilitate expansion, invasion, establishment and persistence for parasites (e.g.,^9,13^). Degree-day models have previously been widely used to understand the broader implications of climate warming on the distributional changes of species^58–62^, however, they are based on fairly simple ecological assumptions and do not incorporate key transmission variables such as transmission rates, population densities, spatial distributions of hosts etc. Increasingly robust models for dynamic transmission populated with parasite life-history traits and host population and movement data will help to further disentangle the complexities associated with the differential patterns of range expansion of these lungworms and in other parasite-host systems (e.g.,^63^).

Empirical data and baselines in conjunction with mechanistic models, provide strong evidence that Arctic warming has led to progressive changes in the distribution of permissive environments and has relaxed thermal constraints, facilitating range expansion, establishment, and persistence of two northern nematode parasites. While annual degree-day (*ADD*) accumulation across our study area generally increased over time, our detailed examination of conditions on Victoria Island demonstrated that this increase was characterized by considerable interannual variability in occurrence of conditions conducive for parasite development. We described a shifting thermal balance over time from years that were historically and predominantly non-permissive for parasite development to years that were predominantly permissive, facilitating invasion, persistence and emergence of the parasites. Thus, the interaction between hosts, parasites, thermal tolerances and thresholds, and fluctuating climatic conditions, is critical in limiting or facilitating the spatial and temporal potential for emerging disease. Habitats and host-parasite assemblages we have explored on Victoria Island are not idiosyncratic. General mechanisms are at play at high latitudes, as evidenced by similar trends in distribution, invasion, host association and explosive emergence of disease for phylogenetically distant filarioid nematodes, with arthropod and vector-borne transmission, among reindeer and other cervids across northern Finland^64,65^.

A wobbling climate over time drives episodes of *environmental sloshing* (recurrent environmental-faunal expansion and contraction) that occurs under alternating regimes of warming and cooling. Sloshing events unfold at varying temporal and spatial scales, from glacial-interglacial cycling of the Quaternary to shallow ecological time such as thermal shifts emerging from the ENSO or PDO and are central to faunal assembly relative to latitude and altitude that shapes the biosphere^12,13^. Changing patterns and amplitudes of warming and cooling influence the continuity of host-parasite systems across evolutionary to ecological time in the Arctic and globally^12,13^. Our observations provide direct insights about the central role of climate drivers in creating recurring opportunities for exploitation of geographical space by parasites^66^. Exemplified are the processes of intercontinental expansion and geographic colonization that characterized the assembly of mosaic parasite faunas linking Eurasia and North America and at intracontinental scales globally during the Pleistocene (e.g.,^12,13^). Contemporary communities, including a protostrongylid fauna with species of *Varestrongylus* and *Umingmakstrongylus,* and a diverse assemblage of other mammalian parasites reflect this intricate history^67^.

Anthropogenic warming now dominates climate conditions in northern systems, with accelerating warming evident since the 1970s. Trajectories for incremental warming in ecological time can be further influenced by decadal scale ocean–atmosphere dynamics and oscillations of the PDO and ENSO which directly influence temperature and environments in terrestrial systems^13,68,69^. Empirical observations from the Central Canadian Arctic in our study, as well as studies from Finland, demonstrate that these short-term shifts in warming and cooling may have important effects on opportunities for invasion that ultimately determine the distribution of pathogens and diseases. Our observations further confirm a general relationship linking capacities for parasites in utilizing host and environmental resources while exploring and exploiting opportunities emerging from ecological disruption which lead to shifting boundaries for distribution^70,71^. These dynamics emerge from ecological fitting and interactions of physical environment (Darwin’s nature of the conditions) and host-parasite biology (nature of the organism) under regimes of changing environmental settings^66^.

Warming is exerting pervasive and cascading environmental effects in the Arctic, and will continue to do so, with numerous and compounding ecological, social, and political perturbations^2,72^. Wildlife remains a critical resource for arctic peoples, foundational to their cultural well-being, and simultaneously an important source of food and income in a landscape where food-insecurity is widespread^10,73^. Recent disease outbreaks^74,75^, local and severe muskox declines^15,76^, widespread declines in caribou^14,77^, and enigmatic mortalities of seals^78^ and a massive shift in the structure of the Bering Sea ecosystem^79,80^, highlight the critical urgency of understanding biotic outcomes of unprecedented climate change and the downstream reverberations in host–pathogen interactions across the Arctic ecosystem.

## Supplementary information

Supplementary information.

## Data Availability

The data that support the findings of this study are available upon request.
