# Supplementary material for: Range expansion of muskox lungworms track rapid arctic warming: implications for geographic colonization under climate forcing
Source: Sci Rep. 2020 Oct 14;10:17323. doi: 10.1038/s41598-020-74358-5 (PMC7560617; doi:10.1038/s41598-020-74358-5)
Supplement: Supplementary file 1 — Supplementary information. [file 41598_2020_74358_MOESM1_ESM.docx]

Supplementary information

Range Expansion of Muskox Lungworms Track Rapid Arctic Warming: Implications for Geographic Colonization Under Climate Forcing

**Authors:**

Pratap Kafle, Peter Peller, Alessandro Massolo, Eric Hoberg, Lisa-Marie Leclerc, Matilde Tomaselli, and Susan Kutz

**Supplementary table 1: Prevalence and intensity of infection of protostrongylid lungworms infecting muskoxen in different areas of the Canadian Arctic based on fecal examination. Samples (n = 1380) were collected between March 2013 and December 2017.**

**Prevalence and intensity of infection of *Umingmakstrongylus pallikuukensis***

|  | In Fig 1. | Area | n | Prev (%) | 95% CI | Median LPG | 95% CI | LPG range | Coinfection^1^ (%) |
| --- | --- | --- | --- | --- | --- | --- | --- | --- | --- |
| Mainland | 1 | Sahtu | 92 | 99${}^{a}$ | 94 - 100 | 215${}^{a}$ | 155.4 - 307.0 | 0.2 - 2458.2 | 8.7 |
|  | 2 | Kugluktuk | 65 | 98${}^{a}$ | 92 - 100 | 149${}^{b}$ | 97.0 - 179.0 | 5.8 - 1669.8 | 52.3 |
|  | 3 | Northern Quebec | 38 | - | - | - | - | - | - |
|  | 4 | Bathurst Inlet | 59 | 64${}^{b}$ | 51 - 76 | 8.7${}^{c}$ | 3.2 - 19.8 | 0.2 - 153.9 | 45.7 |
|  | 15 | Northern Yukon | 18 | - | - | - | - | - | - |
| Victoria Island | 5 | Lady Franklin | 19 | 100${}^{a}$ | 82 - 100 | 182${}^{a}$ | 55.9 - 268.0 | 20.9 - 809.4 | 47.4 |
|  | 6 | Cambridge Bay | 235 | 50${}^{b}$ | 43 - 56 | 4.93${}^{c}$ | 2.9 - 9.0 | 0.2 - 2432.4 | 25.1 |
|  | 7 | Ulukhaktok | 276 | 51${}^{b}$ | 45 - 57 | 8.17${}^{c}$ | 3.2 - 19.8 | 0.2 - 441.0 | 0.7 |
|  | 8 | Central Victoria | 185 | 80${}^{c}$ | 74 - 85 | 5.64${}^{c}$ | 3.7 - 6.8 | 0.2 - 251.4 | 0 |
|  | 9 | Hadley Bay | 69 | 14${}^{d}$ | 7 - 25 | 1.38${}^{d}$ | 0.3 - 10.3 | 0.1 - 68.6 | 0 |
| Other Islands | 10 | Banks Island | 42 | - | - | - | - | - | - |
|  | 14 | King William Island | 40 | - | - | - | - | - | - |
|  | 11 | High Arctic Islands | 230 | - | - | - | - | - | - |

**Prevalence and intensity of infection of *Varestrongylus eleguneniensis***

| Mainland | 1 | Sahtu | 92 | 9${}^{a}$ | 4 - 16 | 22.8${}^{a}$ | 1.8 - 59.6 | 0.8 - 241.4 |  |
| --- | --- | --- | --- | --- | --- | --- | --- | --- | --- |
|  | 2 | Kugluktuk | 65 | 54${}^{b}$ | 41 - 66 | 15.4${}^{a}$ | 7.9 - 20.5 | 0.2 - 269.3 |  |
|  | 3 | Northern Quebec | 38 | 68${}^{b}$ | 51 - 82 | 11.6${}^{a}$ | 7.1 - 15.1 | 0.7 - 90.5 |  |
|  | 4 | Bathurst Inlet | 59 | 54${}^{b}$ | 41 - 67 | 1.12${}^{b}$ | 0.7 - 1.4 | 0.2 - 6.7 |  |
|  | 15 | Northern Yukon | 18 | 56${}^{b}$ | 31 - 78 | 7.4${}^{a}$ | 0.95 - 22.71 | 0.60 - 151.2 |  |
| Victoria Island | 5 | Lady Franklin | 19 | 47${}^{bc}$ | 24 - 71 | 13.1${}^{a}$ | 6.4 - 14.7 | 6.4 - 55.1 |  |
|  | 6 | Cambridge Bay | 235 | 34${}^{c}$ | 28 - 40 | 1.5${}^{b}$ | 0.7 - 3.0 | 0.2 - 90.5 |  |
|  | 7 | Ulukhaktok | 276 | $<$ 1${}^{d}$ | 0 - 3 | 41.2${}^{\#}$ | 7.5 - 41.2 | 7.5 - 74.9 |  |
|  | 8 | Central Victoria | 185 | 35${}^{c}$ | 28 - 42 | 1.4${}^{b}$ | 1.0 - 2.2 | 0.2 - 39.9 |  |
|  | 9 | Hadley Bay | 69 | 0${}^{d}$ | 0 - 5 | - | - | - |  |
| Other Islands | 10 | Banks Island | 42 | - | - | - | - | - |  |
|  | 14 | King William Island | 40 | - | - | - | - | - |  |
|  | 11 | High Arctic Islands | 230 | - | - | - | - | - | - |

**Prevalence and intensity of infection of *Protostrongylus stilesi***

|  | 15 | Northern Yukon | 18 | 83 | 59 - 96 | 7.9 | 3.4 - 23.0 | 0.4 - 283.7 | 44.4 |
| --- | --- | --- | --- | --- | --- | --- | --- | --- | --- |

^1^ Co-infected with *U. pallikuukensis* and *V. eleguneniensis*, except at Northern Yukon where co-infection of *V. eleguneniensis* and *P. stilesi* is present

^#^Represents the average of the two values.

Prevalence of the parasite was quantified as the proportion of the samples having the parasite larvae, the intensity of infection as the median of the larval counts (lpg- larvae per gram) of infected hosts, and abundance as the mean larval counts from all individuals sampled ^1^ . Binomial confidence interval (95%) around the prevalence was determined by ‘exact' method using “binom'' package in R ^2^. To account for the highly skewed distribution of parasite intensity, a 95% confidence interval around the median intensity was determined using the bias-corrected and accelerated (BCa) bootstrapping over 10000 replications, using “boot'' package in R. The range of lpg was also provided. The Fisher exact test, corrected for multiple comparisons, was performed to compare prevalence among locations and between two species. A permutation test, based on 1000 permutations of median lpg, was used to compare the intensity of infections across the locations and between species. Statistical tests for differences in prevalence and intensity of infection between the locations for both species were performed separately. Groups sharing the same letter (a,b, c, d superscripts) are not significantly different. *P* values were adjusted for multiple comparisons.

LPG - larva per gram of feces, Prev – Apparent prevalence, CI – Confidence Interval

**Supplementary fig 1**: Sampling locations and observed distribution of protostrongylids in muskoxen in the Canadian Arctic. The numbers on the map correspond to those values in the second colum of the preceeding table.


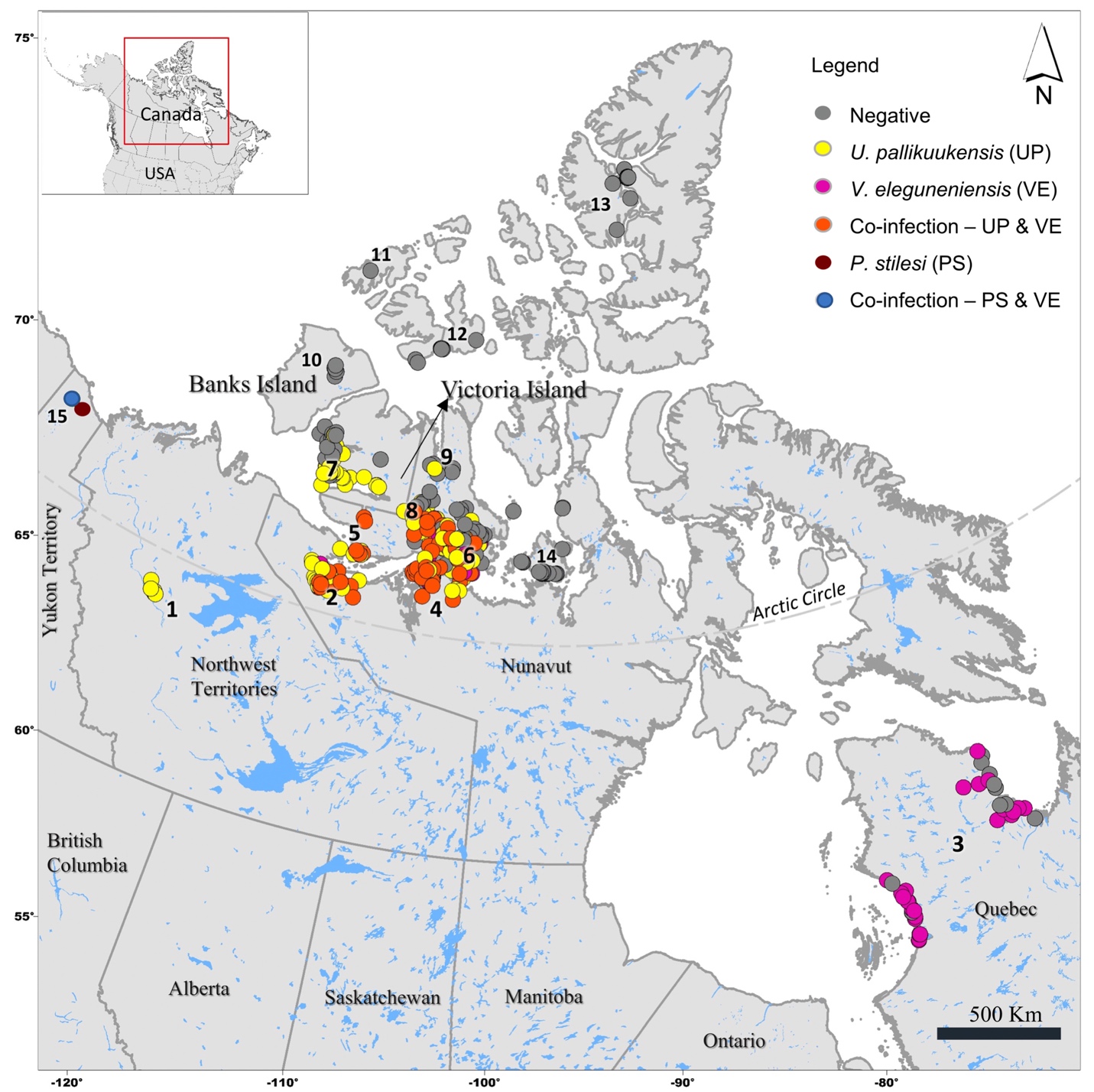


## Supplementary table 2. Yearly prevalence (apparent) and abundance (mean larvae per gram of feces) around Cambridge Bay, NU, Canada.

| **year** | **n** | ***UP* prevalence**  **(95% CI)** | ***UP* abundance**  **(95% CI)** | ***VE* prevalence**  **(95% CI)** | ***VE* abundance**  **(95% CI)** |
| --- | --- | --- | --- | --- | --- |
| 2009 | 182 | 0  (0 - 2) | 0 | 0  (0 - 2) | 0 |
| 2010 | 162 | 0  (0 - 2) | 0 | 4  (2 - 8) | 0.02  (0 - 0.04) |
| 2011 | 94 | 0  (0 - 4) | 0 | 5  (2 - 12) | 0.05  (0 - 0.12) |
| 2012 | 10 | 10  (2 - 40) | 0.03  (0 - 0.1) | 24  (13 - 39) | 0.73  (0.29 - 1.29) |
| 2013 | 39 | 46  (32 - 61) | 8.32  (4.05 - 13.28) | 28  (17 - 44) | 1.34  (0.43 - 2.7) |
| 2014 | 87 | 60  (49 - 69) | 36.35  (15.83 - 61.84) | 34  (25 - 45) | 3.45  (1.39 - 6.4) |
| 2015 | 83 | 37  (28 - 48) | 48.73  (8.23 - 115.01) | 20  (13 - 30) | 0.62  (0.25 - 1.1) |
| 2016 | 20 | 65  (43 - 82) | 36.28  (6.21 - 73.36) | 50  (30 - 70) | 1.64  (0.26 - 3.94) |
| 2017 | 16 | 88  (64 - 97) | 29.2  (3.94 - 73.48) | 81  (57 - 93) | 5.77  (1.06 - 14.37) |

**Supplementary fig 2:** Graph showing the rising prevalence of lungworms around Cambridge Bay, NU.


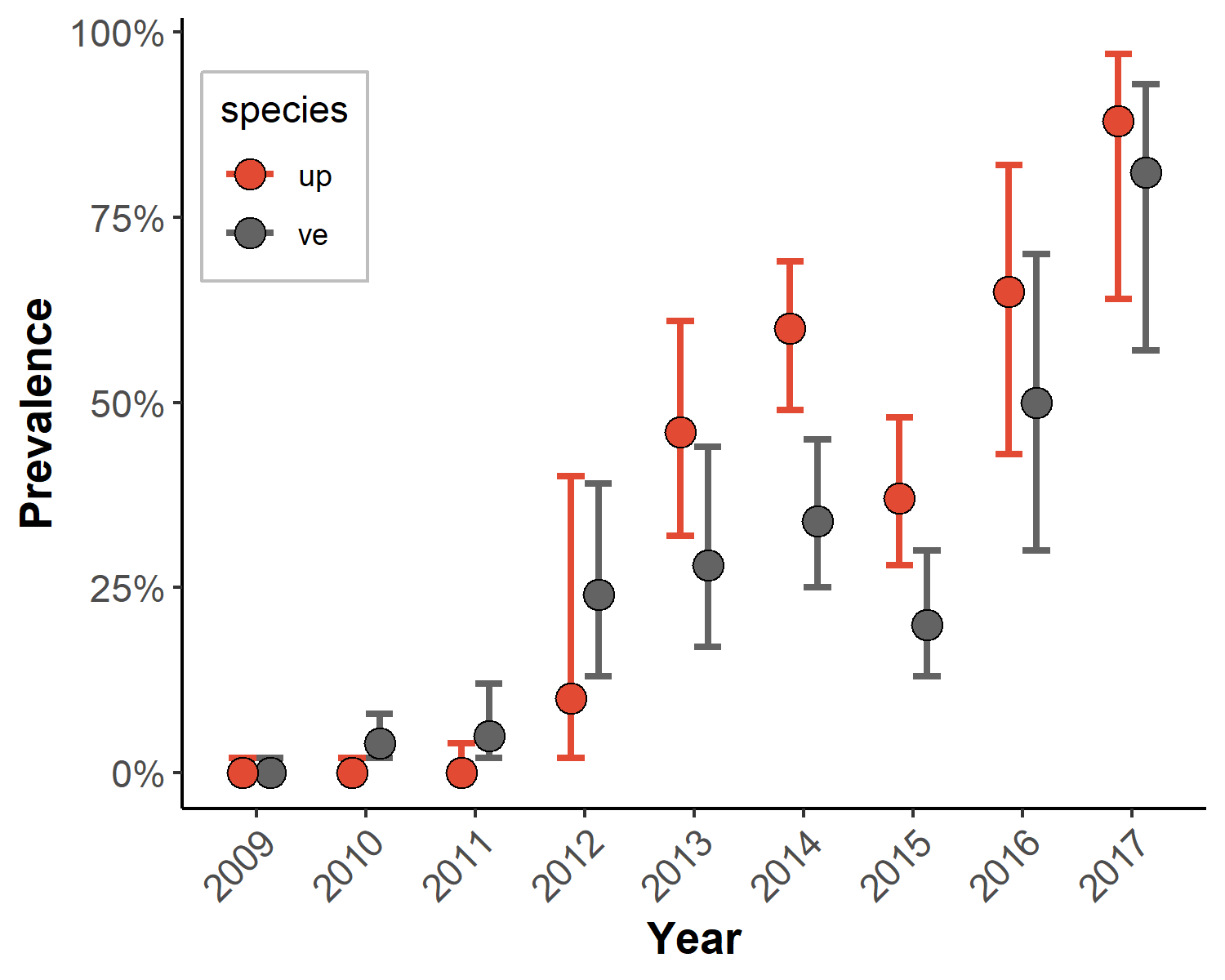


**Supplementary table 3. Summary of the negative binomial model describing the change in intensity of infection of the lungworms over time around Cambridge Bay, NU, Canada.**

Dependent variable: Larvae per 5 grams of feces

Covariates: Year (2009 to 2017), Species (*UP* and *VE*)

|  | Estimate | Std. Error | Z value | P value |
| --- | --- | --- | --- | --- |
| Intercept | -3389 | 121 | -28.1 | < 0.0001*** |
| Year | 1.685 | 0.060 | 28.1 | < 0.0001*** |
| Species *VE* | -0.537 | 0.264 | -2.03 | 0.04* |

Theta = 0.0622 (SE = 0.004)

Null deviance: 1124.17 with 1461 df

Residual deviance: 510.94 with 1459 df

**Supplementary fig 3:** Graph showing the rising intensity of infection of UP and VE around Cambridge Bay, NU, Canada.


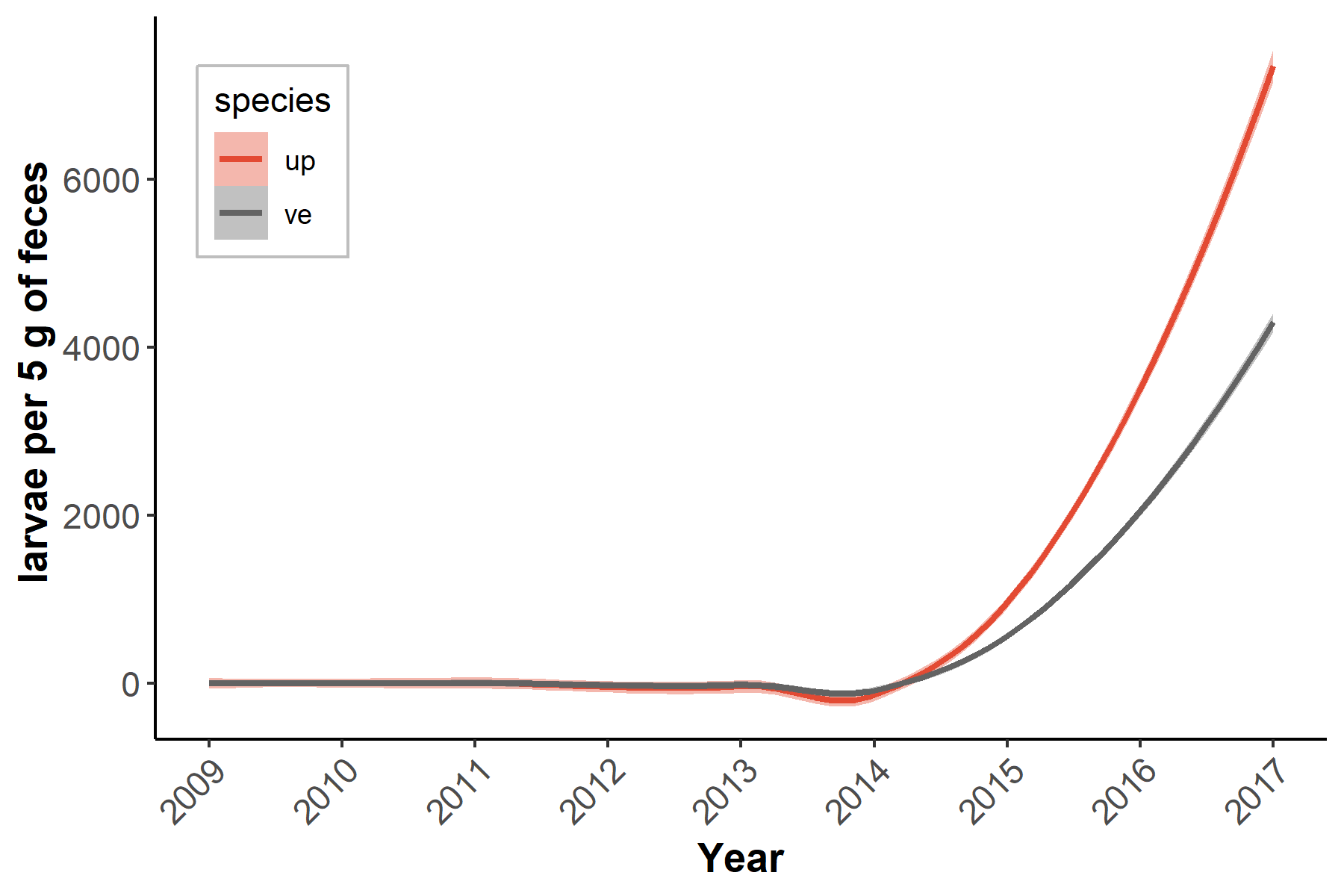


**Supplementary fig 4:** Mean annual temperature (degree C) around Cambridge Bay location from 1980 to 2017. Graph generated by averaging 3 hourly temperature data (2 meter air temperature) from North American Regional Reanalysis. The graph shows a sharp increase in mean annual temperature from around 2000.


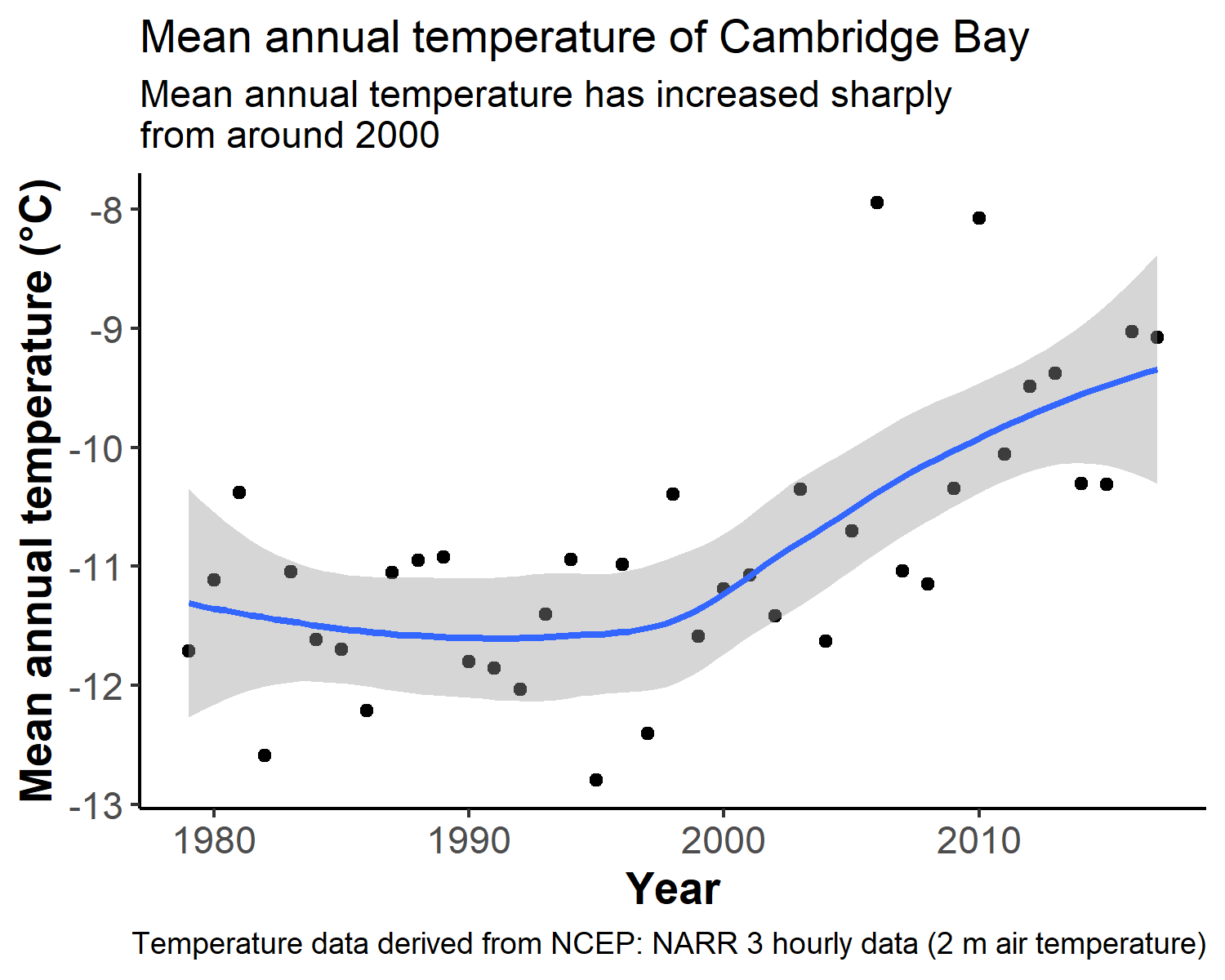


**Supplementary fig 5:** Annual trend in seasonal temperature (degree C) around Cambridge Bay location from 1980 to 2017. The graph shows strong seasonal pattern in warming, with greater rate of winter warming particularly during the last decade. The graph also shows that during the period of 1980 to 2000 mean winter temperature was in decreasing trend, however mean summer temperature was in increasing trend.


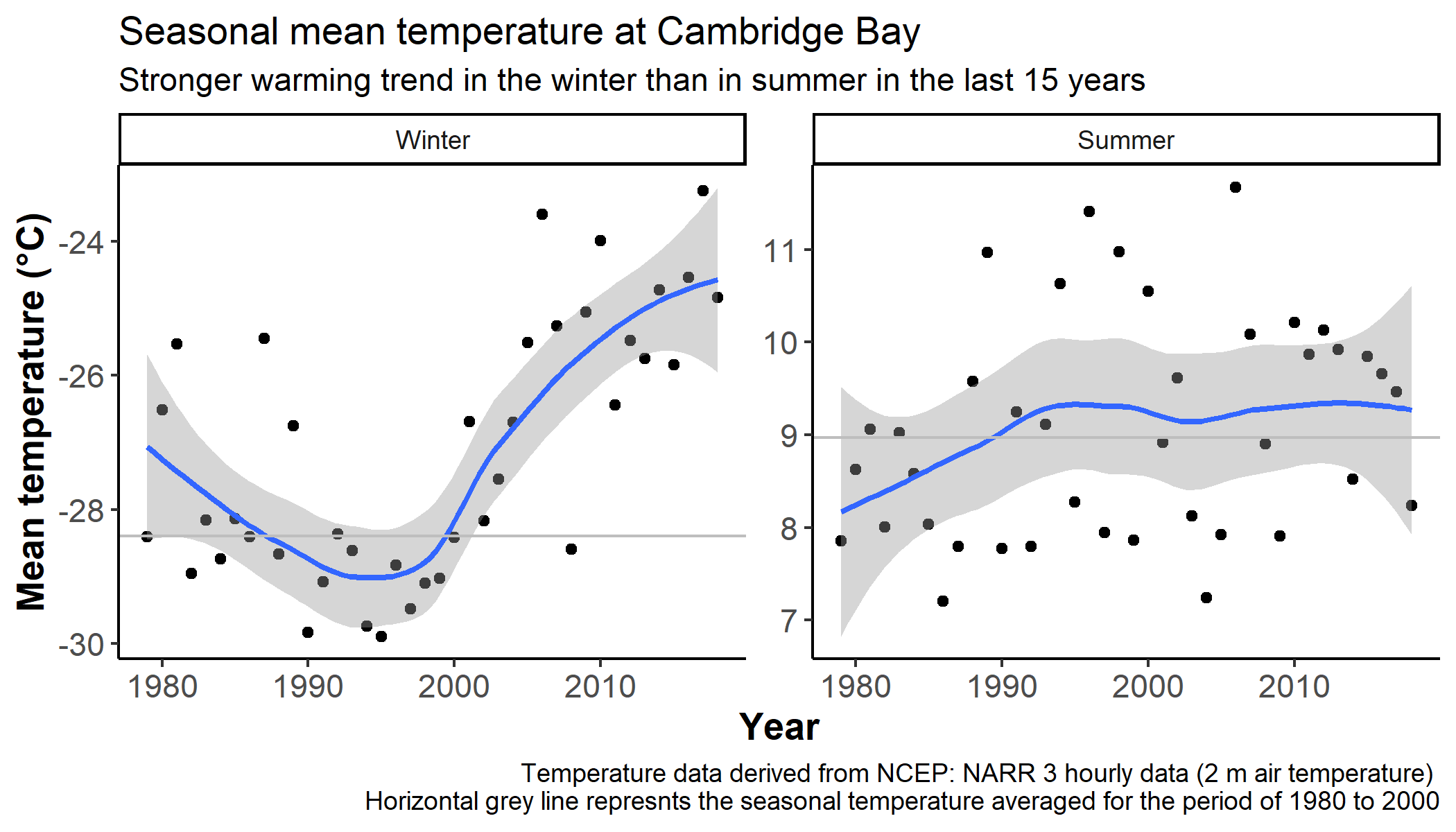


**References**

1. Rózsa, L., Reiczigel, J. & Majoros, G. Quantifying parasites in samples of hosts. *J. Parasitol.* **86**, 228–232 (2000).

2. R Development Core Team. *R: A language and environment for statistical computing*. (R Foundation for Statistical Computing, 2017).
